# Quantitative evaluation of hemodynamic parameters by echocardiography in patients with post-cardiotomy cardiac shock supported by extracorporeal membrane oxygenation

**DOI:** 10.1186/s13019-022-02074-4

**Published:** 2023-01-04

**Authors:** Fuyong Ye, Yuwen Yang, Yinting Liang, Jianhua Liu

**Affiliations:** 1 Department of Medical Ultrasound, Gaozhou People’s Hospital, Gaozhou, Guangdong China; 2 Department of Medical Ultrasound, Guangzhou First People’s Hospital, School of Medicine, South China University of Technology, Guangzhou, China

**Keywords:** Post-cardiotomy cardiac shock, Extracorporeal membrane oxygenation, Hemodynamic, Right heart function

## Abstract

**Background:**

When drugs fail to reverse post-cardiotomy cardiac shock (PCS), extracorporeal membrane oxygenation (ECMO) is considered the most effective adjuvant strategy. Transthoracic echocardiography is a useful imaging modality for monitoring of cardiac hemodynamics. The aim of this study was to investigate the value of echocardiography for monitoring the left and right heart hemodynamics in PCS patients before, during, and after weaning from ECMO.

**Methods:**

Fifty-two patients were divided into successful weaning group (group A, n = 23) and non-successful weaning group (group B, n = 29). Hemodynamic parameters measured by echocardiography were collected before, during, and after ECMO. The intra-group changes and inter-group differences were retrospectively analyzed.

**Results:**

In group A, the central venous pressure (CVP), proximal right ventricular outflow tract (RVOT), tricuspid annular plane systolic excursion (TAPSE), velocity of tricuspid valve (TVDV), and systolic velocity of tricuspid annulus (${\text{s}}_{{{\text{TV}}}}^{{\prime }}$) during ECMO were significantly lower than those before ECMO. After ECMO, left ventricular ejection fraction (LVEF), systolic velocity of mitral annulus (${\text{s}}_{{{\text{MV}}}}^{{\prime }}$), and velocity–time integral of LV outflow tract (LVOT-VTI) were higher than pre-ECMO levels, and CVP, LVEF, ${\text{s}}_{{{\text{MV}}}}^{{\prime }}$, LVOT-VTI, RVOT, TAPSE, TVDV and ${\text{s}}_{{{\text{TV}}}}^{{\prime }}$ were higher than those during ECMO (all *P* < 0.05).

In group B, compared to pre-ECMO, subjects exhibited decreased CVP, RVOT, TAPSE, TVDV and ${\text{s}}_{{{\text{TV}}}}^{{\prime }}$ during ECMO. TAPSE, TVDV, and ${\text{s}}_{{{\text{TV}}}}^{{\prime }}$ were continuously lower after ECMO, while CVP and RVOT increased after ECMO (all *P* < 0.05). After ECMO, LVEF, ${\text{s}}_{{{\text{MV}}}}^{{\prime }}$, LVOT-VTI, TAPSE, TVDV and ${\text{s}}_{{{\text{TV}}}}^{{\prime }}$ in group A were higher than those in group B (all *P* < 0.05). Inter-group comparison showed the LVEF and RV Tei indices of group A were significantly different than those of group B before, during, and after ECMO.

**Conclusion:**

Quantitative assessment of both LV and RV by echocardiography is important for ECMO weaning. Patients with better LVEF and lower RV Tei index may have a better chance of successful weaning from ECMO.

## Introduction

After cardiac surgery, 0.5–1.0% of patients develop refractory cardiogenic shock (CS) [1]. Traditional drug therapy is sometimes inadequate in achieving hemodynamic stabilization in these patients, necessitating temporary mechanical circulatory assistance. Patients receiving cardiac surgery often have a long history of abnormal hemodynamics and severe cardiac decompensation [2–5]. These conditions can lead to severe cardiac dysfunction postoperatively. Veno-arterial extracorporeal membrane oxygenation (V-A ECMO), as a short-term cardiac and respiratory support, is currently one of the first-line treatments for post-cardiotomy cardiogenic shock (PCS) [6–8]. V-A ECMO is considered the most effective support and auxiliary strategy, which can increase arterial pressure and improve blood perfusion in the brain, coronary arteries, and peripheral tissues, and reduce cardiac work and oxygen consumption. This helps gain the time required for recovery of cardiac function and significantly improves the survival rate. Transthoracic echocardiography (TTE) is an important noninvasive imaging modality for monitoring cardiac hemodynamics before, during, and after ECMO. Our hypothesis was that the hemodynamic processes in patients with successful weaning differ from those in patients with unsuccessful weaning. Therefore, in this study, we investigated the dynamic changes in both the left and right heart parameters of patients with PCS who were scheduled for ECMO weaning. The objective was to assess the value of echocardiography in monitoring cardiac hemodynamics of PCS patients before, during, and after weaning from ECMO.

## Material and methods

### Patients

This was a retrospective study. From January 2016 to August 2020, a total of 8556 adult patients underwent cardiac surgery at the cardiothoracic department of the Gaozhou People's Hospital. Among these, 52 (0.6%) patients received ECMO assistance because of unsuccessful weaning from cardiopulmonary bypass (CPB) or refractory PCS [8] and were eligible for the study. Exclusion criteria were: 1) ECMO assistance was used for cardiac postoperative respiratory failure; 2) age < 18 years; 3) deceased patients who did not undergo weaning tests; or 4) patients with incomplete clinical data.

Weaning was successful in 23 patients (group A). However, in 29 patients (group B), total systemic circulatory support and venous oxygen saturation could not achieve the targets, cardiac function did not improve, and weaning was impossible. VA-ECMO support had to be withdrawn, and the patients subsequently died. Six patients in the successful weaning group died after cessation of ECMO (in-hospital death). The causes of death included cerebral hemorrhage (2 patients), multiple cerebral infarction (1 patient), uncontrollable infection (2 patients), and persistent hypotension of unknown cause (1 patient).

This retrospective study was approved by the investigational review board and the requirement for informed patient consent was waived.

### ECMO management

The ECMO system used in this study (Sorin, UK) consisted of centrifugal pumps, polypropylene hollow fiber membrane oxygenator, and heparin-coated tubes. Femoral arteriovenous intubation was performed, and an additional 16 Fr catheter was inserted into the distal femoral artery to avoid distal limb ischemia.

During ECMO assistance, the patient was supported with mechanical ventilation. Midazolam and fentanyl were continuously administered to maintain sedation, and heparin was continuously administered to maintain prothrombin time within the range of 140–180 s.

The blood flow was adjusted according to heart rate, blood pressure, and central venous pressure during ECMO. The pulsatility of cannula on ECMO was observed, and abnormal pulsation was considered indicative of insufficient drainage [9]. Venous oxygen saturation (SvO_2_) was maintained above 0.7. The gas flow rate was calibrated according to the blood flow so as to maintain the ratio of gas flow to blood flow at 0.5–0.8. The respiratory, circulatory, and other clinical indicators were evaluated daily.

Intra-aortic balloon pump (IABP) was added in case of peripheral malperfusion. The indicators included: 1) urine volume < 0.5 mL/kg/h; 2) anaerobic metabolic state or metabolic acidosis (pH < 7.3, blood lactate > 3.0 mmol/L).

### Measurement of echocardiographic parameters

All patients were dynamically monitored for cardiac conditions before, during, and after ECMO using a Philips CX50 ultrasound system (CX50; Philips Ultrasound, Bothell, WA, USA) equipped with an S5-1 transducer (1–5 MHz) integrated with ECG. All echocardiograms were analyzed in accordance with published guidelines [10]. Standard images were collected from parasternal long-axis and short-axis views and the apical four-chamber view. The echocardiographic parameters included left ventricular end-diastolic diameter (LVEDD measured by M-mode), left ventricular ejection fraction (LVEF, measured by biplane Simpson’s method), tissue Doppler imaging (TDI) derived velocity of lateral mitral annulus in systole (${\text{s}}_{{{\text{MV}}}}^{{\prime }}$) and diastole (${\text{e}}_{{{\text{MV}}}}^{{\prime }}$), mitral valve flow Doppler early diastolic velocity (E_MV_), and velocity–time integral at left ventricular outflow tract (LVOT-VTI). Right heart parameters included the dimension of proximal right ventricular outflow tract (RVOT), right ventricular fractional area change (FAC), tricuspid annular plane systolic excursion (TAPSE), right ventricular Tei index and velocity of lateral tricuspid annulus in systole (${\text{s}}_{{{\text{TV}}}}^{{\prime }}$) and diastole (${\text{e}}_{{{\text{TV}}}}^{{\prime }}$) from TDI, and tricuspid valve flow Doppler early diastolic velocity (E_TV_). The same parameters were repeatedly measured, i.e., before establishment of ECMO, when the patient was assisted with maximal flow on the first day on ECMO, and when the ECMO was reduced to a minimal flow for scheduled weaning.

All patients with IABP were at 1:1 support. The IABP was paused at the time of image acquisition. Two ultrasound doctors with more than 10 years of clinical experience independently completed the above measurements.

### Weaning trial

The criteria for the ECMO weaning trial included either not prescribed, or very low dose, vasoactive drugs, mixed venous oxygen saturation (SVO_2_) ≥ 70%, central venous pressure (CVP) < 12 cmH_2_O, mean arterial pressure (MAP) > 60 mmHg, LVEF > 30%, and persisting arterial pulsatility wave on the monitor. ECMO flow was decreased by 0–1 L/min until it reached 1 L/min. Persistence of hemodynamic stability at 1 L/min flow level was considered indicative of successful weaning trial. ECMO was removed when the patient was stable for an additional 15 min under monitoring after stoppage of the ECMO flow.

### Statistical analysis

SPSS20.0 was used for statistical analysis. Normality of distribution of continuous variables was assessed using the Shapiro–Wilk test. Normally-distributed continuous variables were expressed as mean ± standard deviation and between-group differences were assessed using the Student's *t* test. Categorical variables were presented as frequencies and percentages and between-group differences were assessed using the Chi-squared or Fisher’s exact test. Comparison of hemodynamic parameters between the two groups at different stages (before, during, and after ECMO) was performed using two-way repeated measure analysis of variance. *P* values less than 0.05 were considered indicative of statistical significance.

## Results

### Comparison of the baseline characteristics between the two groups (Table 1)

**Table 1:** Baseline characteristics of the study population and clinical outcomes

Parameter	Group A (n = 23)	Group B (n = 29)	*P* value
Age(y)	55.3 ± 15.9	62.1 ± 10.6	*t* = − 1.834, *P* = 0.154
Male sex	12 (52.2%)	21 (72.4%)	*x*^2^ = 2.266, *P* = 0.132
BMI (kg/m^2^)	29.0 ± 8.5	31.0 ± 10.3	*t* = − 0.729,* P* = 0.470
Hypertension	5 (21.7%)	11 (37.9%)	*x*^2^ = 1.579,* P* = 0.209
Hyperlipemia	9 (39.1%)	7 (24.1%)	*x*^2^ = 1.353,* P* = 0.245
Diabetes	1 (4.3%)	5 (17.2%)	*x*^2^ = 1.017,* P* = 0.313
PAH	4 (17.4%)	16 (55.2%)	*x*^2^ = 6.222,* P* = 0.013
Arrhythmia	1 (4.3%)	4 (13.8%)	*x*^2^ = 0.454,* P* = 0.500
Currently smoking	1 (4.3%)	1 (3.4%)	*x*^2^ = 0.000,* P* = 1.000
Previous myocardial infarction	6 (26.1%)	8 (27.6%)	*x*^2^ = 0.015,* P* = 0.904
History of cardiac surgery	1 (4.3%)	2 (6.9%)	*x*^2^ = 0.000,* P* = 1.000
NYHA Class I-II	12 (52.2%)	17 (58.6%)	*x*^2^ = 0.216,* P* = 0.642
NYHA Class III-IV	11 (47.8%)	12 (41.4%)	*x*^2^ = 0.216,* P* = 0.642
CABG	6 (26.1%)	11 (37.9%)	*x*^2^ = 0.818,* P* = 0.366
Valvular surgery	7 (30.4%)	14 (48.3%)	*x*^2^ = 1.696,* P* = 0.193
CABG + Valvular surgery	2 (8.7%)	1 (3.4%)	*x*^2^ = 0.043,* P* = 0.836
Operation of CHD	2 (8.7%)	1 (3.4%)	*x*^2^ = 0.043,* P* = 0.836
Operation of aorta	6 (26.1%)	2 (6.9%)	*x*^2^ = 2.304,* P* = 0.129
Emergency operation	9 (39.1%)	14 (48.3%)	*x*^2^ = 0.435,* P* = 0.510
CPB time (min)	343.9 ± 136.1	276.2 ± 134.1	*t* = 1.796,* P* = 0.079
Cross-clamp time (min)	295.7 ± 114.9	238.2 ± 131.9	*t* = 1.649,* P* = 0.105
Albumin (g/L)	38.4 ± 5.7	39.5 ± 7.8	*t* = − 0.551,* P* = 0.584
Total bilirubin (μmol/L)	41.6 ± 26.5	39.3 ± 14.0	*t* = 0.395,* P* = 0.695
Serum creatinine (μmol/L)	171.7 ± 43.8	185.3 ± 57.6	*t* = − 0.971,* P* = 0.336
Prothrombin time (s)	22.0 ± 6.2	25.0 ± 14.8	*t* = − 0.920,* P* = 0.362
Lactate dehydrogenase (IU/L)	222.2 ± 53.8	230.6 ± 41.0	*t* = − 0.623,* P* = 0.537
NT-proBNP (μmol/L)	2042.0 ± 539.1	1881.9 ± 691.7	*t* = 0.911,* P* = 0.367
IABP	11 (47.8%)	4 (13.8%)	*x*^2^ = 5.675,* P* = 0.017
CRRT	5 (21.7%)	5 (17.2%)	*x*^2^ = 0.167,* P* = 0.683
Dopamine (μg/kg/min)	5.44 ± 0.89	5.93 ± 0.98	*t* = − 1.861, *P* = 0.069
Adrenaline (μg/kg/min)	0.19 ± 0.10	0.25 ± 0.14	*t* = − 1.619,* P* = 0.112
Noradrenaline (μg/kg/min)	0.12 ± 0.04	0.14 ± 0.05	*t* = − 1.995,* P* = 0.052

Data presented as mean ± standard deviation or frequency (percentage)

*AF* atrial fibrillation, *BMI* body mass index, *CABG* coronary artery bypass grafting, *CHD* congenital heart disease, *CPB* cardiopulmonary bypass, *CRRT* continuous renal replacement therapy, *IABP* intra-aortic balloon pump, *NT-proBNP* N-terminal pro-brain natriuretic peptide, *NYHA* New York Heart Association functional class, *PAH* pulmonary artery hypertension

There were no significant between-group differences with respect to age, sex, body mass index, comorbidities, New York Heart Association (NYHA) class, type of operation, or preoperative biochemical examination (*P* > 0.05).

The percentage of patients with pulmonary artery hypertension (PAH) in group B [16 patients (55.2%)] was significantly greater than that in group A [4 patients (17.4%); *P* < 0.05]. The percentage of patients with intra-aortic balloon pump (IABP) support in group A [11 patients (47.8%)] was significantly greater than that in group B [4 patients (13.8%); *P* < 0.05].

### Comparison of the echocardiographic and hemodynamic parameters under different ECMO conditions (Table 2; Figs. 1, 2, 3, 4 and 5)

**Table 2:** Hemodynamic and echocardiographic parameters of the two groups under different ECMO conditions

Parameter	Group A (n = 23)			Group B (n = 29)		
	Pre-ECMO	During ECMO	After ECMO	Pre-ECMO	During ECMO	After ECMO
Flow rate (L/min)		4.3 ± 0.8			3.9 ± 0.8	
HR (bpm)	81.7 ± 15.3	85.9 ± 12.9	79.8 ± 9.9	82.9 ± 11.4	87.8 ± 12.1	85.1 ± 16.7
MAP (mmHg)	74.1 ± 7.1	87.4 ± 15.1^a^	84.1 ± 9.7^a^	77.7 ± 6.8	89.0 ± 12.7^a^	72.7 ± 15.5^bc^
StO_2_ (%)	76.6 ± 8.4	94.7 ± 12.2^a^	93.7 ± 9.2^a^	76.6 ± 8.7	78.7 ± 9.0^c^	73.5 ± 8.2^c^
CVP (mmHg)	10.6 ± 2.2	6.5 ± 2.8^a^	9.5 ± 3.2^b^	9.7 ± 2.0	7.9 ± 2.7^a^	10.1 ± 3.5^b^
LVEDD (mm)	49.9 ± 10.0	50.5 ± 9.9	52.0 ± 11.0	53.1 ± 10.1	53.6 ± 7.8	50.2 ± 7.9
LVEF (%)	31.4 ± 8.1	30.4 ± 6.8	36.4 ± 11.3^ab^	26.8 ± 7.3^c^	24.7 ± 6.3^c^	28.3 ± 7.4^c^
${\text{s}}_{{{\text{MV}}}}^{{\prime }}$(cm/s)	4.7 ± 0.7	4.8 ± 0.6	6.2 ± 0.5^ab^	4.7 ± 0.7	4.8 ± 0.8	4.5 ± 0.7^c^
E_MV_/${\text{e}}_{{{\text{MV}}}}^{{\prime }}$	7.4 ± 2.6	8.8 ± 2.6	7.6 ± 2.4	7.4 ± 2.6	8.2 ± 2.6	9.8 ± 3.3
LVOT-VTI (cm)	10.2 ± 2.7	8.6 ± 3.1	13.9 ± 3.8^ab^	9.2 ± 3.0	8.6 ± 2.0	8.9 ± 2.3^c^
RVOT-prox (mm)	29.1 ± 3.9	25.7 ± 2.9^a^	29.1 ± 4.3^b^	33.1 ± 6.9	25.1 ± 4.1^a^	30.7 ± 4.5^b^
FAC (%)	26.3 ± 9.8	24.9 ± 7.2	27.6 ± 8.8^b^	24.6 ± 8.6	22.3 ± 7.8	24.0 ± 10.0
TAPSE (mm)	12.2 ± 2.0	11.4 ± 3.2^a^	14.6 ± 2.3^b^	13.7 ± 3.7	10.6 ± 2.5^a^	10.6 ± 2.3^ac^
TVDV (cm/s)	43.3 ± 9.4	32.3 ± 9.4^a^	49.1 ± 10.0^b^	42.4 ± 9.4	34.2 ± 9.5^a^	36.7 ± 8.2^ac^
${\text{s}}_{{{\text{TV}}}}^{{\prime }}$(cm/s)	10.0 ± 1.6	6.5 ± 1.7^a^	10.4 ± 1.7^b^	8.9 ± 2.5	7.0 ± 1.4^a^	7.5 ± 1.7^ac^
Tei index	0.43 ± 0.10	0.47 ± 0.12	0.43 ± 0.11	0.57 ± 0.15^c^	0.59 ± 0.21^c^	0.57 ± 0.17^c^
E_TV_/${\text{e}}_{{{\text{TV}}}}^{{\prime }}$	5.9 ± 1.8	6.0 ± 2.2	5.4 ± 1.9	5.8 ± 1.8	5.3 ± 2.2	5.3 ± 1.4

*CVP *central venous pressure, *E*_*MV*_ early diastolic flow velocity at mitral valve, ${\text{e}}_{{{\text{MV}}}}^{{\prime }}$ early diastolic velocity of mitral annulus, *E*_*TV*_ early diastolic flow velocity at tricuspid valve, ${\text{e}}_{{{\text{TV}}}}^{{\prime }}$ early diastolic velocity of tricuspid annulus, *FAC* fractional area change, *HR* heart rate, *LVEDD* left ventricular end-diastolic diameter, *LVEF* left ventricular ejection fraction, *LVOT-VTI* velocity-time integral of left ventricular outflow tract, *MAP* mean arterial pressure, *RVOT-prox* proximal right ventricular outflow tract, *StO*_*2*_ peripheral tissue oxygen saturation, ${\text{s}}_{{{\text{MV}}}}^{{\prime }}$ velocity of mitral annulus in systolic, ${\text{s}}_{{{\text{TV}}}}^{{\prime }}$ velocity of tricuspid annulus in systolic, *TAPSE* tricuspid annular plane systolic excursion

^a^Intra-group comparisons: pre-ECMO versus during ECMO or after ECMO, *P* < 0.05

^b^During ECMO versus after ECMO, *P* < 0.05

^c^Inter-group comparisons: Group A veruss Group B at the same ECMO state, *P* < 0.05

**Fig. 1:**
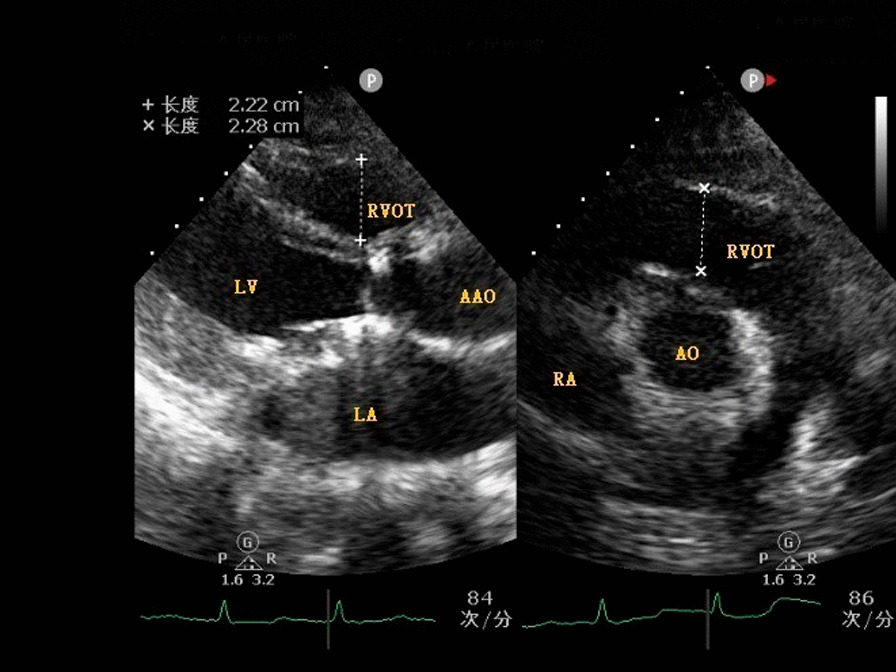
Proximal right ventricular outflow tract (RVOT-prox)

**Fig. 2:**
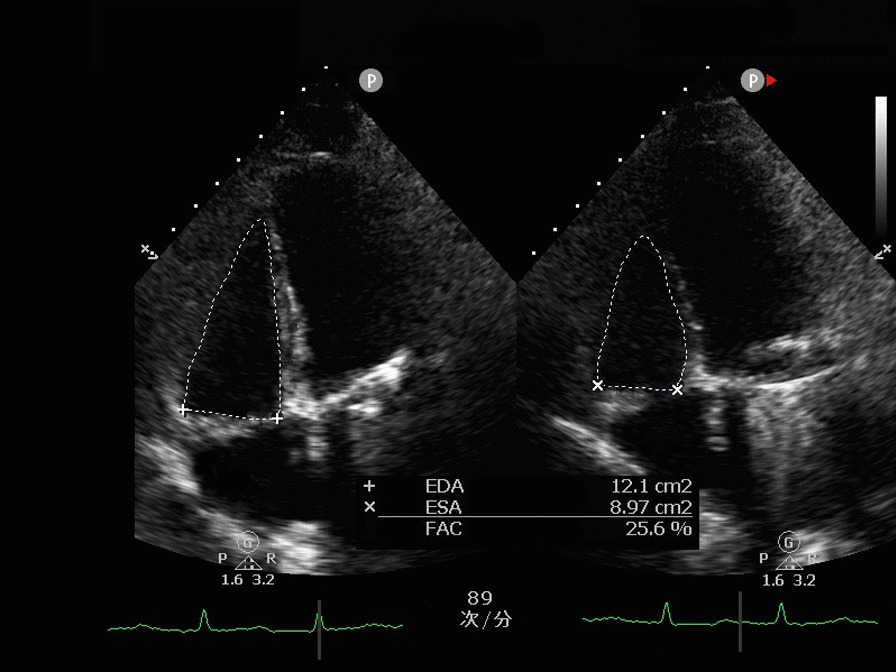
Right ventricular fractional area change (FAC)

**Fig. 3:**
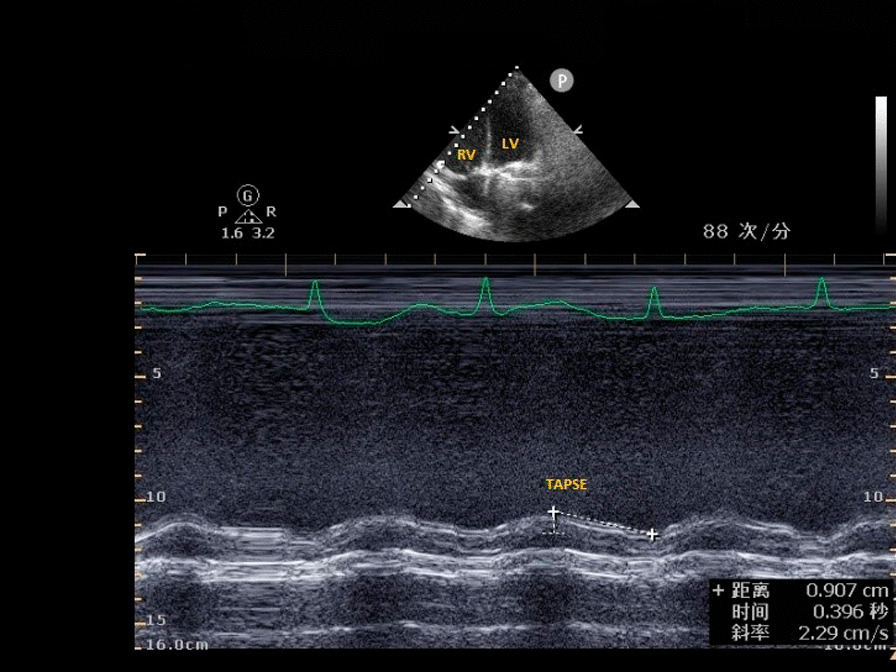
Tricuspid annular plane systolic excursion (TAPSE)

**Fig. 4:**
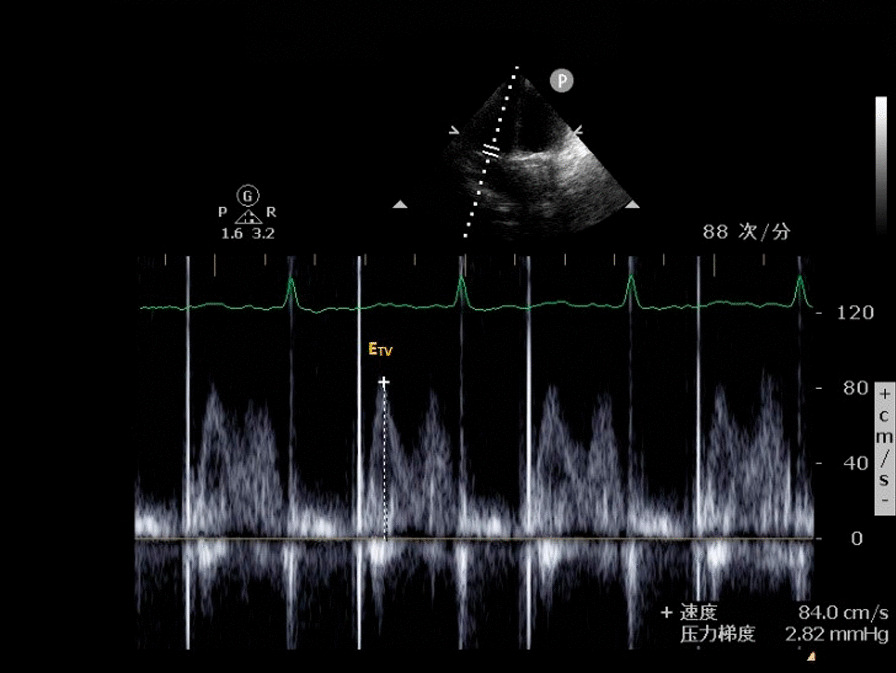
Early diastolic velocity of tricuspid valve (E_TV_)

**Fig. 5:**
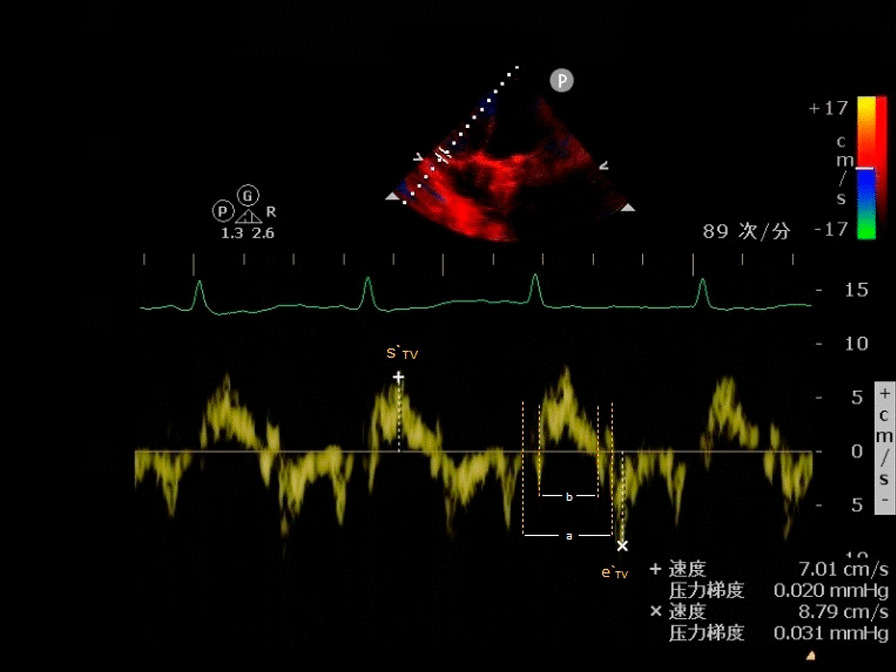
Tei index [= (a − b)/b], velocity of tricuspid annulus in systolic (${\text{s}}_{{{\text{TV}}}}^{{\prime }}$) and early diastolic velocity of tricuspid annulus (${\text{e}}_{{{\text{TV}}}}^{{\prime }}$)

#### Group A intra-group comparison

Compared with pre-ECMO, mean arterial pressure (MAP) and peripheral tissue oxygen saturation (StO_2_) were significantly higher on the first day of ECMO (*P* < 0.05), while central venous pressure (CVP), RVOT, TAPSE, TVDV, and ${\text{s}}_{{{\text{TV}}}}^{{\prime }}$ were significantly lower (*P* < 0.05).

After ECMO, the values of MAP, StO2, LVEF, ${\text{s}}_{{{\text{MV}}}}^{{\prime }}$, and LVOT-VTI were higher compared with pre-ECMO levels (*P* < 0.05), while CVP, LVEF, ${\text{s}}_{{{\text{MV}}}}^{{\prime }}$, LVOT-VTI, RVOT, TAPSE, TVDV, and ${\text{s}}_{{{\text{TV}}}}^{{\prime }}$ were higher than that during ECMO (*P* < 0.05).

#### Group B intra-group comparison

Compared to pre-ECMO, subjects in the unsuccessful weaning group exhibited increased MAP and decreased CVP, RVOT, TAPSE, TVDV, and ${\text{s}}_{{{\text{TV}}}}^{{\prime }}$ on the first day of ECMO (*P* < 0.05), while TAPSE, TVDV and ${\text{s}}_{{{\text{TV}}}}^{{\prime }}$ were significantly lower after ECMO (*P* < 0.05).

Compared to during ECMO, subjects exhibited lower MAP, but higher CVP and RVOT after ECMO (*P* < 0.05).

In contrast to group A, in group B, the pre-ECMO parameters, including MAP, StO_2_, LVEF, ${\text{s}}_{{{\text{MV}}}}^{{\prime }}$ and LVOT-VTI, were not significantly different compared with those after ECMO (*P* > 0.05).

### Inter-group comparison of echocardiographic and hemodynamic parameters before, during, and after weaning from ECMO

Before ECMO, the LVEF in group A was significantly higher than that in group B. On the first day of ECMO, StO_2_ and LVEF in group A were significantly higher than those in group B. After ECMO, the hemodynamic parameters of group A, including MAP, StO_2_, LVEF, ${\text{s}}_{{{\text{MV}}}}^{{\prime }}$, LVOT-VTI, TAPSE, TVDV, and ${\text{s}}_{{{\text{TV}}}}^{{\prime }}$, were higher than those in group B. The right ventricular Tei indices of group A were significantly lower than those of group B before, during, and after ECMO. The differences were statistically significant (*P* < 0.05).

## Discussion

In this study, patients with successful weaning showed significant improvement in the left ventricular systolic function during ECMO, while the right ventricular systolic function initially declined followed by its restoration to the pre-ECMO level. In contrast, patients with unsuccessful weaning continued to remain in a low cardiac output state, and their right ventricular systolic function decreased and failed to recover.

ECMO has been shown to facilitate myocardial recovery in patients with PCS. Studies have shown that ECMO helps achieve good long-term outcome in these patients but is associated with a high in-hospital mortality [5, 11–14]. The current study included 52 adult patients who received VA-ECMO for PCS, among which 29 patients had unsuccessful weaning and subsequently died. There were 6 additional deaths (in-hospital) after successful weaning from ECMO. The hospital mortality rate was 67.3%. The successful weaning rate and survival rate in this study were consistent with studies conducted overseas [15–18].

PCS patients usually receive VA-ECMO, in which venous blood is elicited, CO_2_ is removed from venous blood, the blood is fully oxygenated, and then injected into the aorta [19, 20]. Patients connected to the ECMO undergo a series of hemodynamic changes. Management of ECMO primarily focuses on regulating the flow, which is essential for the circulatory system, and at the same time preventing higher left ventricular diastolic pressure. Insufficient flow rate cannot improve peripheral circulatory failure and cardiogenic shock. Conversely, excessively high flow rate increases the left ventricular end-diastolic filling pressure, left atrial pressure, and pulmonary capillary wedge pressure, and may result in pulmonary edema. The high flow may also affect aortic valve opening, which further aggravates myocardial damage.

In the baseline analysis, the parameters that significantly differed between the successful weaning group and unsuccessful weaning group included IABP support and PAH. The heart of PCS patient sustains a double insult from cardiac surgery and cardiopulmonary bypass. Studies have shown that the combination of IABP and ECMO helps reduce left ventricular afterload and increases coronary blood supply, which promotes myocardial recovery. In addition, the application of IABP can reduce pulmonary edema and increase cerebral blood supply. Theoretically, the combined application of IABP and ECMO should have better auxiliary effect [4, 21–24]. The results of this study were consistent with this theory.

Heart disease may lead to secondary pulmonary hypertension characterized by pathological changes such as pulmonary intimal fibrosis, thickening of intima media, and necrotizing arteritis. However, pulmonary vasodilators have limited effect in this setting. Even after resolution of the primary cause of pulmonary hypertension, pulmonary artery hypertension may persist in these patients, which worsens the prognosis [25, 26]. Another serious negative effect of pulmonary hypertension is impairment of right ventricular function. Progressive increase in the pulmonary artery resistance increases the right ventricular afterload, leading to hypertrophy of right ventricular cardiomyocytes. The increase in myocardial oxygen consumption in the right ventricle is accompanied by a relative insufficiency of blood supply, which gradually impairs the right ventricular systolic function. On the other hand, decreased compliance of the thickened right ventricle also impairs the diastolic function [27].

Our study included various echocardiographic parameters. Ultrasound is suitable for bed-side monitoring of hemodynamic and functional changes at any time, and is superior to many other diagnostic modalities in this respect.

Ultrasound is indispensable for monitoring and evaluation before, during, and after ECMO. First, cardiac echocardiography plays a key role in clinical decision-making by assessing the indication/contraindication for ECMO. Second, echocardiography can help guide the intubation and setting up of ECMO. Echocardiography allows for real-time monitoring of the changes in cardiac cavity size, volume, and function, enabling regulation of the ECMO flow to balance the peripheral perfusion and left ventricular diastolic pressure. Finally, echocardiography is a useful tool in guiding the ECMO weaning.

In our study, there was no significant difference between the two groups with respect to the flow rate on the first day during ECMO. The MAP in the two groups increased significantly after the establishment of the VA-ECMO. This was attributable to the withdrawal of a large amount of venous blood into the circular cannula, which is liable to decrease the cardiac preload. However, significant improvement in StO_2_ was only observed in the successful weaning group, which may be due to more severe microcirculation disorder in the non-successful weaning group. Because of the preload reduction, the decreases in the CVP, RVOT, TAPSE, ${\text{s}}_{{{\text{TV}}}}^{{\prime }}$, and TVDV in both groups during the ECMO support were expected, and the differences in these parameters between the two groups were not significant. The echocardiographic parameters that showed a significant difference at baseline (before ECMO) between the two groups were LVEF and Tei index, which indicates that pre-ECMO LV systolic function and RV function may help predict the success of ECMO treatment. However, it is technically challenging to determine the Tei index in patients who are required to be in supine position after cardiac surgery; thus, this index is liable to considerable interobserver variability. Therefore, when evaluating the RV function, other parameters of RV, including FAC, TAPSE, ${\text{s}}_{{{\text{TV}}}}^{{\prime }}$, E_TV_/${\text{e}}_{{{\text{TV}}}}^{{\prime }}$ should be comprehensively considered.

Another key use of echocardiography is to guide ECMO weaning. After weaning from ECMO, the increase in CVP and RVOT in both groups was due to a large amount of blood returning to the right heart and lung circulation. This led to increase in the cardiac preload and disappearance of the blood retrograde pressure, leading to decreased afterload. Neither group showed significant changes in LVEDD. The effect of increase in the afterload during ECMO, which can cause LV dilation, may be partly offset by the decrease in the preload. According to Platts et al. [28], recovery of left ventricular systolic function is the key to successful weaning from ECMO. They used LVOT-VTI > 10 cm, LVEF > 20%–25% and Sa > 6 cm/s at the minimum flow rate as the weaning criteria. They found that the accuracy of these parameters in judging the success of weaning was significantly better than other parameters. In our study, the MAP, StO _2_, LVEF, LVOT-VTI, and ${\text{s}}_{{{\text{MV}}}}^{{\prime }}$ of the successful weaning group at the minimum flow were significantly higher than those before ECMO establishment. Additionally, we found that the right ventricular systolic functional parameters (TAPSE, ${\text{s}}_{{{\text{TV}}}}^{{\prime }}$) of the two groups changed differently when at the minimum flow. The right ventricular function of the successfully weaned patients returned to pre-ECMO levels, while the unsuccessfully weaned patients continued to show decreased performance. In the intergroup comparison, TAPSE, ${\text{s}}_{{{\text{TV}}}}^{{\prime }}$, MAP, StO2, LVEF, LVOT-VTI, and ${\text{s}}_{{{\text{MV}}}}^{{\prime }}$ were significantly higher in the successfully weaned group. This indicates that, in the unsuccessfully weaned group, the myocardial damage in both left and right ventricles could not recover or be further aggravated by ECMO treatment. Based on the above findings, we believe the recovery of right ventricular systolic function is also an important echocardiographic indication that cannot be ignored in ECMO weaning [29].

### Limitations

This was a single-center retrospective study, which may have introduced an element of bias. It was previously reported that Tei index is paradoxically decreased in severe RV dysfunction with increased RA pressure. This shortcoming of RV Tei index may cause a misjudgment of RV function [30]. Even with the device being paused at the time of imaging, the addition of an IABP in a proportion of the study population may have interfered with the hemodynamic data due to temporal changes in the loading conditions.

## Conclusion

Transthoracic echocardiography (TTE) plays an indispensable role in the monitoring of cardiac hemodynamics in patients undergoing ECMO. Thanks to its simple operation and non-invasive characteristics, TTE is ideal for bedside monitoring of real time changes in cardiac hemodynamics. Accurate understanding of the changes in hemodynamic parameters is essential for maximal efficacy of ECMO. Therefore, quantitative evaluation of hemodynamic parameters is of great significance during ECMO therapy.

## Data Availability

The datasets used and analyzed during the current study are available from the corresponding author on reasonable request.
